# Manifestation of Pancytopenia Associated with COVID-19 as Paroxysmal Nocturnal Hemoglobinuria (PNH) and Aplastic Anemia (AA)

**DOI:** 10.3390/hematolrep16010005

**Published:** 2024-01-17

**Authors:** Jeff Justin Aguilar, Vikram Dhillon, Suresh Balasubramanian

**Affiliations:** 1 School of Medicine, Wayne State University, Detroit, MI 48201, USA; ho8226@wayne.edu; 2 Department of Oncology, Karmanos Cancer Institute, Wayne State University, Detroit, MI 48201, USA; vdhillon@med.wayne.edu

**Keywords:** paroxysmal nocturnal hemoglobinuria, aplastic anemia, SARS-CoV-2

## Abstract

We report two cases of pancytopenia in patients after recovering from a mild COVID-19, now presenting as paroxysmal nocturnal hemoglobinuria (PNH) and aplastic anemia. These cases illustrate a common pathway whereby a viral trigger causes the clonal expansion of a hematological disorder. Although the association of both cases with COVID-19 is temporal and COVID-19 may be an incidental diagnosis, the growing evidence related to the hematological effects of SARS-CoV-2 infection highlights the need for further investigation into the hematological consequences of COVID-19, particularly in the post-pandemic era.

## 1. Introduction

COVID-19, caused by the severe acute respiratory syndrome coronavirus 2 (SARS-CoV-2), has caused more than 6.8 million deaths globally as of 22 March 2023 [1]. There are known associations of hematological disorders with other viruses from the Coronaviridae family, such as SARS-CoV-1 and Middle East Respiratory Syndrome coronavirus (MERS-CoV) [2]. Similarly, SARS-CoV-2 has been linked to a broad spectrum of hematological abnormalities, ranging from mild leukopenia to fulminant bone marrow failure. More recently, acquired bone marrow failure syndromes, including aplastic anemia and paroxysmal nocturnal hemoglobinuria, have been reported in the literature as a sequela of COVID-19 infection [3,4,5,6].

Aplastic anemia is a rare hematologic disorder characterized by pancytopenia due to marrow aplasia. The most common etiology of aplastic anemia is immune mediated, and 80% of these cases respond remarkably well to immunosuppressive therapy [7]. The mechanism behind immune-mediated aplastic anemia is not fully understood; however, there are culminating evidence points on the role of viral infections that lead to dysregulated immune responses and the destruction of hematopoietic stem cells (HSCs) [7].

PNH is an acquired disorder of HSCs characterized by somatic mutations in the glycosylphosphatidylinositol (GPI) anchor protein synthesis, which leads to a deficiency of complement regulatory proteins and unregulated complement-mediated hemolysis [7,8,9]. More often, AA and PNH can present in the same patient as a moving target in the same spectrum.

Though it is etiologically challenging to prove the cause of AA/PNH, it is imperative to report this rare condition in association with the recent SARS-CoV2 pandemic. Here, we describe two cases of aplastic anemia and PNH presenting as pancytopenia associated with SARS-CoV-2.

## 2. Methods

This report involves two cases of acquired pancytopenia associated with COVID-19 manifesting as PNH and AA. Data sources were retrospective and collected from the electronic medical record system, including patient histories, labs and diagnostics, the documentation of clinical course, therapies used, and clinical outcomes at follow-up.

## 3. Case Series

### 3.1. Case 1

A previously healthy 21-year-old male presents to the hematology clinic with pancytopenia that was discovered during the pre-operative evaluation of an ankle open reduction and internal fixation (ORIF) in April 2022. His only medical history is that of mild COVID-19 infection confirmed by PCR, and he has no family history of any hematological disorders. Initial labs revealed a hemoglobin of 9.3 g/dL, white blood cell count of 4.1 × 10^3^ cells/μL, absolute neutrophil count of 2.74 × 10^3^ cells/μL, and platelet count of 34 × 10^3^ cells/μL. His coagulation profile (PT/INR, APTT), D-dimer, and fibrinogen levels were within reference range. COVID-19 assessed using nasopharyngeal polymerase chain reaction (PCR) was negative. Hemolytic labs revealed an LDH of 595 U/L and an elevated total bilirubin of 1.4 mg/dL. Bone marrow biopsy showed a decreased trilineage hematopoiesis, but no blasts. Further cytogenetic studies showed an abnormal 13q deletion in 2 out of 20 cells. PNH flow cytometry identified PNH clones in granulocytes (19.53%), monocytes (19.77%), and RBCs (3.61%; 0.22% type II cells and 3.39% type III cells). Since the patient was previously healthy without a family history of hematological disease nor a constellation of symptoms suggesting a possible congenital cause of bone marrow failure, such as Fanconi anemia and dyskeratosis congenita, further genetic testing was not performed. A diagnosis of hemolytic paroxysmal nocturnal hemoglobinuria/non-severe aplastic anemia combination syndrome was made. He was administered the appropriate immunization for encapsulated organisms prior to starting him on ravulizumab. Five months after diagnosis, and after completing three doses of ravulizumab, he showed improvements in counts and did not require transfusions since diagnosis.

### 3.2. Case 2

A 52-year-old female presents to the hematology clinic with increased bruising. She has a medical history of anterior uveitis and fibromyalgia, however, no family history of hematological disorders. She contracted a mild COVID-19 infection in May 2022, confirmed with PCR, and finished a course of nirmatrelvir–ritonavir (Paxlovid) without experiencing any respiratory compromise. A month following her recovery, she experienced easy bruising that prompted a visit to her primary care physician where basic labs were run, and she was found to be pancytopenic. On exam, she did not have any active bleeding, petechiae, abdominal pain, or melena. Notably, she completed her initial vaccination series for SARS-CoV-2 with Johnson & Johnson/Janssen vaccines in 2021, and seven months later, she had a follow-up vaccination with a Pfizer–BioNtech booster. The initial laboratory studies revealed a hemoglobin of 11.6 mg/dL, white blood cell count of 3.0 × 10^3^ cells/μL, absolute neutrophil count of 0.9 × 10^3^ cells/μL, and platelet count of 18 × 10^3^ cells/μL. Bone marrow biopsy revealed decreased trilineage hematopoiesis and no increase in blasts; however, rare, small irregular lymphoid aggregates composed of small mature T and B lymphocytes were present. Flow cytometry revealed normal immunophenotypic results. Next generation sequencing and cytogenetic studies did not reveal any abnormalities, but PNH flow cytometry identified minute PNH clones in FLAER- and CD157-negative neutrophils and monocytes (0.5%). The patient was diagnosed with non-severe aplastic anemia with a non-hemolytic subclinical PNH clone and was managed with observation and a close monitoring of blood counts. She did not develop any further easy bruising or bleeding episodes during observation.

The clinical characteristics of both cases are summarized below in Table 1.

## 4. Discussion

Epidemiological studies estimate the annual incidence of AA in 2019 to be around 2.0 per million in Western countries and higher in Asia (3.0–5.0 per million) [8]. Comparatively, the annual incidence for PNH in the US is 5.7 per million [9]. PNH can affect any age group; however, in the US, the most affected age group is between third and fifth decades. At the time of writing, the incidence rates of AA and PNH in the post-COVID-19 era were unknown. Several studies have reviewed the hematologic manifestations of COVID-19, and the most frequently observed laboratory findings include lymphopenia, neutrophilia, anemia, and thrombocytopenia [2,10,11]. However, as we reported here, some patients have a predilection towards acquiring bone marrow failure syndromes after a COVID-19 infection. A brief compilation of such cases is presented here in Table 2. Although their association with SARS-CoV-2 infection seems temporal and, in some instances, incidental, given the wide prevalence of the virus, an increasing number of new AA and PNH cases related to COVID-19 warrant further exploration of a viral trigger that leads to marrow failure as a sequela. 

This emerging relationship between COVID-19, AA, and PNH is being investigated globally. A survey from the UK examined the emergence of AA in patients recovering from COVID-19 infection and discovered 3 cases of AA (diagnosed as severe or very severe AA) developed a few weeks after a positive SARS-CoV-2 result, 2 cases of AA relapse (confirmed with marrow hypocellularity), and 15 cases of hematologic decline in known AA that required treatment, transfusion support, and monitoring [15]. Additionally, there has been an increase in the number of cases reporting the exacerbation of PNH with COVID-19 infection since the start of the pandemic. Iannuzzi et al. reported 14 cases of known PNH patients and 7 cases of AA/PNH patients who presented with worsening hemolysis symptoms following an infection with SARS-CoV-2 [14]. This underscores the need to include the worsening clinical spectrum in estimating the true incidence of AA/PNH during the COVID-19 pandemic.

**Figure 1:**
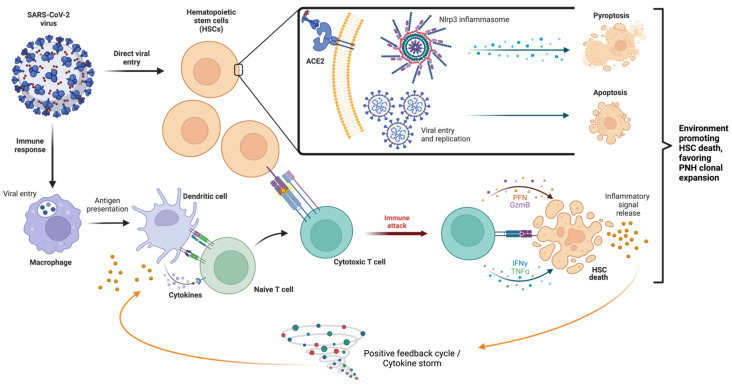
Mechanisms of SARS-CoV-2 on hematopoietic cells. The SARS-CoV-2 virus has two primary mechanisms: direct viral entry (above, inset) and through inciting an immune response (below). Direct viral entry, via ACE2 receptors, may upregulate Nlrp3 inflammasome expression, leading to pyroptosis, viral replication within HSCs, and apoptosis. Immune response causes the activation of T cells that respond to HSCs and subsequent immune attack, leading to the proliferation of inflammatory signals and a cytokine storm. HSC: hematopoietic stem cell; ACE2: angiotensin-converting enzyme 2; and Nlrp3: NOD-like receptor family pyrin domain containing 3. Different color circles represent cells and the corresponding labels of each cell are given in the figure.

A few mechanisms have been proposed regarding the effect of SARS-CoV-2 on the hematopoietic system including the direct effects of the virus on the bone marrow, cytokine storm causing immune-mediated damage, and the direct effect of virus on erythroid precursors [2]. A summary of these mechanisms is illustrated below in Figure 1. Viral infections, such as SARS-CoV-1, MERS-CoV, and SARS-CoV-2, have been shown to induce a pro-inflammatory state wherein activated white blood cells and cytokines are released in a positive feedback loop [8]. Because of this background inflammatory state, immune system hyperactivation results in multiorgan dysfunction [16]. Ratajczak and Kucia (2020) evaluated the role of the Nlrp3–inflammasome complex in generating an inflammatory microenvironment, how the innate immune system interacts with the inflammasome, and the secretion of pro-inflammatory cytokines in COVID-19 and HSCs (Figure 1) [17]. Their research demonstrated the overexpression of inflammasome complex in HSCs during an active infectious period and that SARS-CoV-2 virus entry receptor angiotensin-converting enzyme 2 (ACE2) is also expressed on the surface of HSCs [17]. They hypothesized that SARS-CoV-2 may be involved in the direct transcription of pro-inflammatory mediators in HSCs by a spike protein interaction with the ACE2 receptor, and it may also induce uncontrolled Nlrp3 inflammasome expression, leading to hematopoietic stem cell death via pyroptosis [17,18]. 

The pro-inflammatory microenvironment generated by COVID-19 also plays a role in the immune-mediated acquired aplastic anemia mechanism. The development of abnormal autoimmune responses, including cytotoxic T-cells that activate, expand, and circulate as oligoclones, causes the release of myelosuppressive cytokines and induces the cell death of HSCs and progenitor cells [19]. Nevertheless, the inciting antigens for such a T-cell response remain undetermined, and HLA polymorphisms and the aberrant expression of T-cell receptor signaling genes may also play a role in T-cell dysfunction [20]. Whether the SARS-CoV-2 virus can act as an inciting antigen remains to be clarified.

PNH clonal expansion is found in almost 50% of patients with immune-mediated acquired aplastic anemia [20]. Clonal expansion can be from intrinsic (such as PNH cells being conferred an intrinsic growth advantage via acquired mutations) or extrinsic (such as an environment targeting the destruction of normal HSCs giving rise to PNH HSC selection) in nature. It must be noted that both mechanisms can also co-occur simultaneously [7]. It has also been proposed that PNH cells have acquired the capacity to evade apoptotic stimuli and inflammatory cytokines and escape the HSC-directed immune attack as found in aplastic anemia [20].

**Figure 2:**
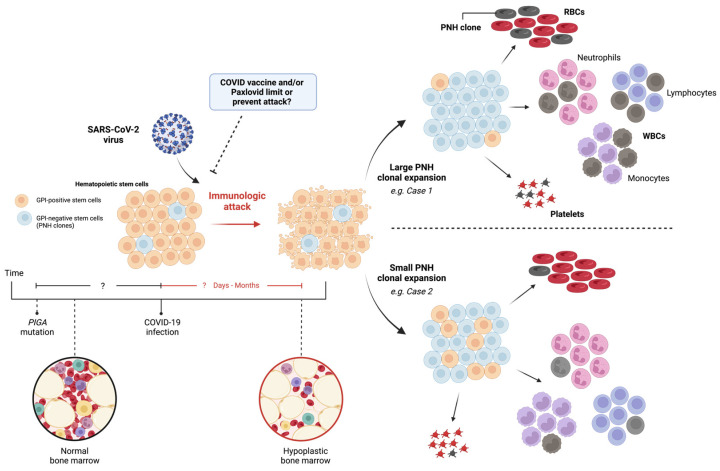
Implications of SARS-CoV-2 virus: immunologic attack and PNH clonal expansion. Normal bone marrow (black circle), with or without PIGA mutation, encounters SARS-CoV-2 virus, leading to immunologic attack and hypoplastic bone marrow (red circle). Direct or indirect mechanisms may cause HSC death, resulting in variable PNH clonal expansion and bone marrow hypoplasia. RBC: red blood cell; WBC: white blood cell; PNH: paroxysmal nocturnal hemoglobinuria; GPI: glucose phosphate isomerase; and PIGA: phosphatidylinositol N-acetylglucosaminyltransferase subunit A. Different color circles represent cells and the corresponding labels of each cell are given in the figure.

Moreover, if SARS-CoV-2 catalyzes an immunologic attack against bone marrow progenitors, these cases illustrate the variability in the virus’s ability to cause PNH clonal expansion and foster an autoimmune marrow environment (Figure 2). Here, case 1 demonstrated a larger PNH clone population, leading to the hemolytic presentation, and case 2 was found to have a small amount of PNH clones. Although the reason for this variability can be multifactorial, a reduced immunologic attack should correspond to the lower levels of PNH clone expansion [20]. 

Based on treatment data reported in the literature, only one patient was treated for the combined COVID-19 infection and PNH with a short course of pulse steroids followed by IVIG (Table 2). However, the investigators noted no hematological improvement and even the use of eculizumab yielded a poor response [3]. In our report, case 2 received two prophylactic COVID-19 vaccines and completed a five-day course of Paxlovid after COVID-19 exposure, whereas case 1 received neither. To compare the clinical characteristics and impact on disease trajectory, we need longitudinal follow-ups and larger retrospective studies [15]. Similarly, data on overall survival (OS) stratified by concomitant COVID-19 and PNH compared to COVID-19 alone are sparsely reported. Median OS appears to be reduced in the combined setting [15]; however, larger studies are needed to better understand how COVID-19 infection influences the overall survival for PNH/AA. Whether the temporal relationship between somatic PIGA mutation and immunologic attack (i.e., COVID-19 infection) determines the degree of clone expansion as well as the effects of COVID vaccination and/or antiviral treatment remain open questions that need exploring in future studies.

## 5. Conclusions

In summary, we report two cases of new-onset PNH/aplastic anemia syndrome and aplastic anemia associated with COVID-19. It is possible that SARS-CoV-2 does play a role in the development of PNH clonal expansion and bone marrow failure, but the exact mechanism is still unknown. Regardless, aplastic anemia is a common ground, perhaps stimulating the formation of clonal populations. Although the association of both cases with COVID-19 is temporal, and COVID-19 may be an incidental diagnosis, the growing evidence related to the hematological effects of SARS-CoV-2 infection highlights the need for further investigation of this phenomenon.

## Figures and Tables

**Table 1:** Clinical information for patients with SARS-CoV-2-related aplastic anemia.

Variable		Patient Case	
		1	2
Age		21	52
Sex		Male	Female
Interval between COVID infection and pancytopenia		4 months	1 month
CBC	WBC	4.1	3.0
	ANC	1.2	0.9
	Platelets	34	18
	HGB	9.3	11.6
	MCV	106.2	94
Bone Marrow Biopsy Cellularity		10–20%	10–15%
PNH clones	Granulocytes	19.53%	N/A
	Monocytes	19.77%	0.5%
	RBCs	3.61%	N/A
History of autoimmune disease		None	Anterior uveitis
SARS-CoV-2 Vaccination		None	Johnson and Johnson/Janssen Seven months later, Pfizer–BioNtech booster
Treatment		Ravulizumab	N/A

**Table 2:** Cases of SARS-CoV-2-related aplastic anemia/PNH.

Clinical Data	Diagnosis	Bone Marrow Biopsy Cellularity	Onset of Cytopenia after COVID Infection	AA/PNH Therapy	COVID-19 Treatment	Reference(s)
21, M *	Hemolytic PNH/aplastic anemia	10–20%	4 months	Ravulizumab	Supportive	This report
52, F *	Aplastic anemia (with subclinical PNH clones)	10–15%	1 month	Observation	Paxlovid	This report
35, M	Hemolytic PNH	Normal	0 days ^‡^	Eculizumab, transfusion support	3-day course of pulse steroids, and IVIG	[3]
22, F	Severe aplastic anemia	5%	10 days	Sibling HSCT	Supportive	[4]
69, F	Severe aplastic anemia	5–10%	2 days	Cyclosporine, antithymocyte globulin, eltrombopag	Supportive	[4]
76, M	Pure red cell aplasia	20–30%	4 months	Cyclosporine	Supportive	[4]
21, M	Severe aplastic anemia (with subclinical PNH clones)	<5%	<1 month	Cyclosporine, antithymocyte globulin, eltrombopag, eculizumab ^†^	Supportive	[4]
69, F	Severe aplastic anemia (with subclinical PNH clones)	5%	5 months	Cyclosporine, antithymocyte globulin, eltrombopag	Supportive	[4]
19, F	Hemolytic PNH	40–50% ^§^	0 days ^‡^	Eculizumab, Ravulizumab	Supportive	[5]
28, F	Severe aplastic anemia	20–30%	3 months	Cyclosporine, antithymocyte globulin, eltrombopag, prednisone	Supportive	[4,6]
21, M	Severe aplastic anemia	<5%	2 months	Sibling HSCT	Supportive	[12]
12, F	Severe aplastic anemia	10%	0 days ^‡^	Antithymocyte globulin, cyclosporine	Paxlovid	[13]
18, M	Severe aplastic anemia	10%	0 days ^‡^	Antithymocyte globulin, cyclosporine	Supportive	[13]
78, F	Hemolytic PNH/aplastic anemia	Poor	0 days ^‡^	-- ^||^	Supportive	[14]

* Our case report. ^†^ Received eculizumab due to expansion of PNH clone. ^‡^ SARS-CoV-2 PCR was positive on presentation; timing between infection and cytopenia onset unclear. ^§^ Biopsy also showed large areas less than 10% cellular. ^||^ Patient was voluntarily discharged prior to the start of any treatment; follow-up unknown.

## Data Availability

The data used to support the findings of this study are included in the article.
